# User Experiences of the NZ COVID Tracer App in New Zealand: Thematic Analysis of Interviews

**DOI:** 10.2196/26318

**Published:** 2021-09-08

**Authors:** Alexei Tretiakov, Inga Hunter

**Affiliations:** 1 School of Management Massey University Palmerston North New Zealand

**Keywords:** COVID-19, contact tracing, app, New Zealand, adoption, use, civic responsibility, privacy

## Abstract

**Background:**

For mobile app–based COVID-19 contact tracing to be fully effective, a large majority of the population needs to be using the app on an ongoing basis. However, there is a paucity of studies of users, as opposed to potential adopters, of mobile contact tracing apps and of their experiences. New Zealand, a high-income country with western political culture, was successful in managing the COVID-19 pandemic, and its experience is valuable for informing policy responses in similar contexts.

**Objective:**

This study asks the following research questions: (1) How do users experience the app in their everyday contexts? and (2) What drives the use of the app?

**Methods:**

Residents of New Zealand’s Auckland region, which encompasses the country’s largest city, were approached via Facebook, and 34 NZ COVID Tracer app users were interviewed. Interview transcripts were analyzed using thematic analysis.

**Results:**

Interviews ranged in duration from 15 to 50 minutes. Participants ranged in age from those in their late teens to those in their early sixties. Even though about half of the participants identified as White New Zealanders of European origin, different ethnicities were represented, including New Zealanders of South Pacific, Indian, Middle Eastern, South American, and Southeast Asian descent. Out of 34 participants, 2 (6%) identified as Māori (Indigenous New Zealanders). A broad range of careers were represented, from top-middle management to health support work and charity work. Likewise, educational backgrounds ranged broadly, from high school completion to master’s degrees. Out of 34 participants, 2 (6%) were unemployed, having recently lost their jobs because of the pandemic. The thematic analysis resulted in five major themes: perceived benefits, patterns of use, privacy, social influence, and need for collective action. Benefits of using the app to society in general were more salient to the participants than immediate health benefits to the individual. Use, however, depended on the alert level and tended to decline for many participants at low alert levels. Privacy considerations played a small role in shaping adoption and use, even though the participants were highly aware of privacy discourse around the app. Participants were aware of the need for high levels of adoption and use of the app to control the pandemic. Attempts to encourage others to use the app were common, although not always successful.

**Conclusions:**

Appeals to civic responsibility are likely to drive the use of a mobile contact tracing app under the conditions of high threat. Under the likely scenario of COVID-19 remaining endemic and requiring ongoing vigilance over the long term, other mechanisms promoting the use of mobile contact tracing apps may be needed, such as offering incentives. As privacy is not an important concern for many users, flexible privacy settings in mobile contact tracing apps allowing users to set their optimal levels of privacy may be appropriate.

## Introduction

### Background

Contact tracing is a nonpharmaceutical intervention commonly used for curbing the spread of COVID-19 [1,2]. Manual contact tracing, conducted by interviewing patients diagnosed with the disease to identify their close contacts, is rather slow, and digital contact tracing involving digitally recording information about individuals’ movements has been suggested to be potentially considerably more effective based on simulation evidence [3]. Mobile apps that perform digital contact tracing have been implemented in many countries, such as Australia [4] and Singapore [5]. Based on cross-country comparison, Urbaczewski and Lee [6] asserted that mobile app–based contact tracing is effective in helping countries to keep COVID-19 under control.

For mobile app–based contact tracing to be fully effective, a large majority of the population needs to be using the app on an ongoing basis [3,7]. The importance of mobile contact tracing app adoption prompted several empirical studies. Trang et al [8] conducted an experiment in Germany where they suggested alternative designs and made different appeals about the benefits offered, asking the respondents to rate their intent to install the app. They found that citizens can be divided into three categories: critics, undecided, and advocates. Critics were more likely to accept an app based on an appeal that by using it they would protect society in general, and they were more likely to accept app designs with strong privacy features. Undecided citizens, similar to critics, responded to societal-benefit appeals, but valued convenience in app use more than they valued privacy. Neither critics nor undecided citizens cared about the app offering health benefits to them as individuals. Finally, advocates responded to both societal-benefit and self-benefit appeals and did not care about privacy or convenience.

In a similar experiment conducted in the United Kingdom, Wiertz et al [9] offered citizens four configurations of an app differing by self-benefits offered, privacy, and the entity overseeing the app. Citizens—treated as a single group—preferred an app offering self-benefits and that was overseen by an independent entity, rather than by the government. Wiertz et al [9] found no evidence suggesting that citizens valued the privacy features of an app. Jonker et al [10] conducted a discrete choice experiment in the Netherlands, allowing citizens to rate a range of possible features of a mobile contact tracing app. Citizens preferred an app that would store data locally and give them control over whether to share it with the authorities. Further, they preferred an app that would offer a small financial reward. Thus, the results regarding the effects of privacy features and of self-benefits were not consistent across studies.

Walrave et al [11] conducted a survey in Belgium to determine factors affecting citizen intention to adopt a mobile contact tracing app. The study used the uniﬁed theory of acceptance and use of technology (UTAUT) framework [12]; performance expectancy (ie, benefits offered by the app, conceptualized by Walrave at al [11] as societal benefits), effort expectancy, social influence, and facilitating conditions (ie, having the knowledge and resources necessary to use the app) were considered as potential factors. Further, innovativeness, privacy concerns, and COVID-19–related stress were added to the basic UTAUT model. The most important factor was performance expectancy, followed by facilitating conditions and social inﬂuence. Innovativeness and privacy concerns had weaker effects on intention to adopt. The results by Walrave et al [11] are complemented by a survey by Altmann et al [13] that was conducted in France, Germany, Italy, the United Kingdom, and the United States. Regarding reasons to install a mobile contact tracing app, the respondents rated benefits to family and friends higher than benefits to the broader community. In addition, they rated concerns about government surveillance and security highly regarding the main reasons against installation, with concerns about government surveillance rated the highest. Further, greater trust in the government was associated with higher app installation intent.

In all the studies introduced above, the participants had no exposure to a real mobile contact tracing app and answered questions with a hypothetical app in mind.

### Research Questions

The results of the studies published so far are not entirely consistent regarding factors driving the adoption of a mobile contact trading app. Further, studies of users, as opposed to potential adopters, of mobile contact tracing apps and their experiences are not available.

Better understanding of user experiences with the NZ (New Zealand) COVID Tracer app is of practical interest for countries using mobile contact tracing apps to protect their populations from COVID-19, particularly if COVID-19 becomes endemic [14], possibly necessitating the continued use of contact tracing over the long term. Further, understanding user experiences with a mobile contact tracing app is of broader theoretical interest for epidemiology. Therefore, this study asks the following research questions:

1. How do users experience the app in their everyday contexts?
2. What drives the use of the app?

## Methods

### Overall Approach and Study Setting

Qualitative design was used, as it is particularly suitable for an exploratory study of user experiences [15-17]. Data were collected via semistructured interviews with users of the NZ COVID Tracer app, New Zealand’s official mobile contact tracing app overseen by the Ministry of Health [18].

The study was conducted in the Auckland region; Auckland is the biggest city in New Zealand. New Zealand’s COVID-19 outbreak response, as assessed in October 2020, has been recognized as successful [19]. Thus, the New Zealand experience may be of interest. The Auckland region was chosen because it has experienced more COVID-19–related disruption than the rest of the country, as detailed in the following section.

Interviews were conducted 5 months after the app became available, allowing us to explore user experiences in the context of how the pandemic situation in the Auckland region and the functionality of the app evolved over time. This context is described in the following section.

### NZ COVID Tracer App and COVID-19 Pandemic in New Zealand

The NZ COVID Tracer app was released by New Zealand’s Ministry of Health on May 20, 2020 [20], simultaneously for iOS and for Android platforms. The app was presented as a “digital diary,” allowing the recording of places the users of the app visited by scanning QR (Quick Response) codes. Users could also register their contact details with the app to make it easier for COVID-19 contact tracers to reach them.

Privacy was emphasized in the app design and in the Ministry of Health’s communications about the app: information about places visited by the user was stored locally on the phone and was not shared with contact tracing services automatically; in the initial release of the app, the user had to open the app and read out the information to contact tracers. Further, the information was automatically deleted after 31 days. Moreover, for security and privacy reasons, to use the app users had to log on using a strong (ie, sufficiently long and complex) password. The password had to be re-entered every 30 days, resulting in confusion for some of the users, who would not remember the password and, thus, were locked out of the app, as documented in comments on the Apple App Store [21] and on Google Play [22].

On June 15, 2020, the app was updated to allow users to be notified if they visited a venue around the same time as a known COVID-19 case [23]. This feature was implemented without sending users’ location data to the Ministry of Health. Further, users could now send their location data to contact tracers if they chose to do so. If the initial version of the app solely supported the contact tracing process, thus offering benefits for the community or for the country as a whole, the updated app offered immediate benefits to the users, who, in case of exposure, could be diagnosed earlier and could receive early treatment, thus improving their prognosis [24]. Moreover, users receiving an alert could self-isolate, thus protecting their family, friends, and colleagues.

Benefits to the community, the user’s family, and the user as an individual have been repeatedly highlighted in subsequent communications by the Ministry of Health: “Taking a few seconds to scan in with the app means we can quickly inform you when you may have been exposed to the virus, so you can take steps to protect yourself and your whānau [extended family],” “It also means if you test positive for the virus, you can instantly provide your digital diary to contact tracers to give them a massive head-start,” and “The faster we can contact trace, the quicker we can get ahead of the virus and prevent spread in the community” [25].

Another major update of the app was on July 30, 2020, when the ability to add manual entries to record visits to locations with no QR codes, such as visits to friends and family, was added [26], allowing one “to maintain a complete – and private – record.” Initially, organizations were encouraged but not required to display QR codes compatible with the Ministry of Health’s NZ COVID Tracer app [20]. However, starting from August 19, 2020, displaying QR codes became compulsory for most business premises and for many transport services [27].

**Figure 1:**
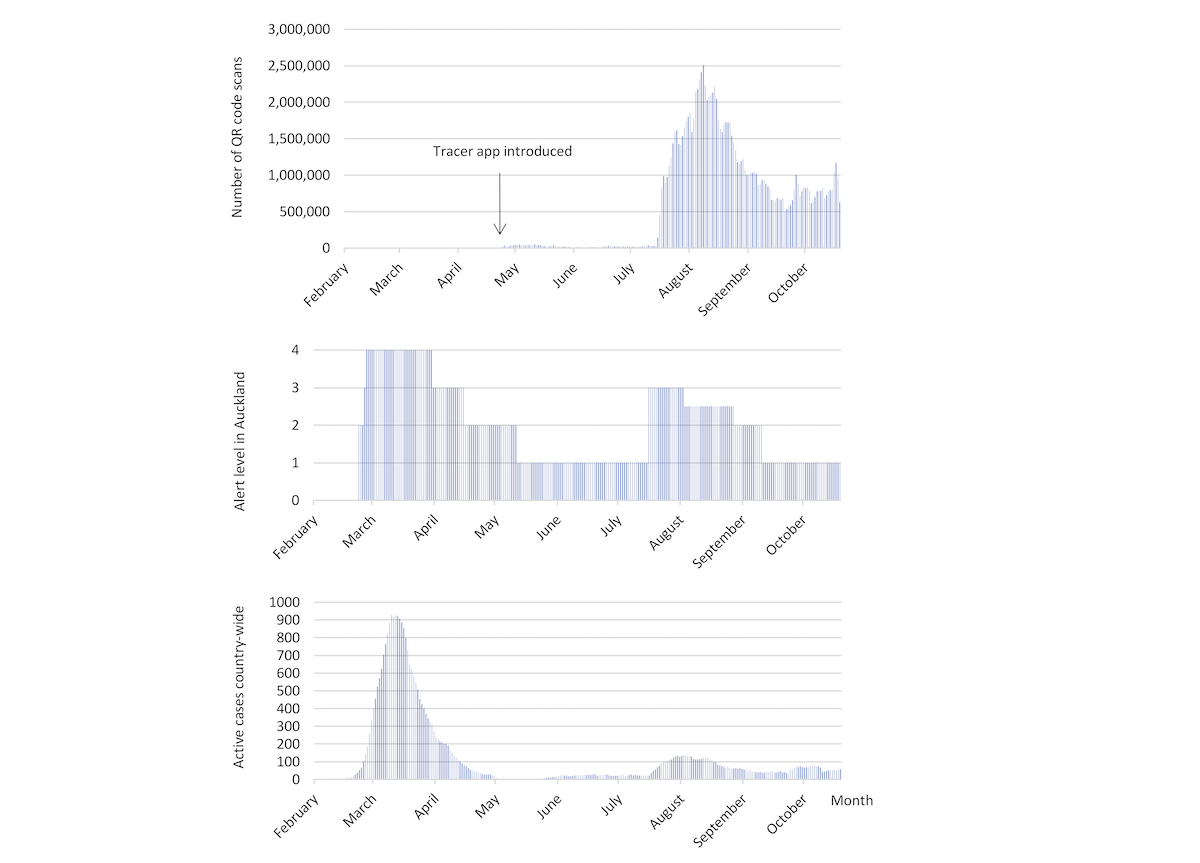
COVID-19 pandemic in New Zealand and NZ COVID Tracer app use in 2020. QR: Quick Response.

Even though location data were held locally on users’ phones, usage data, including the number of app registrations, the number of active devices, the number of QR code scans, and the number of manual entries, were available to the Ministry of Health, and some aggregate data were routinely shared via media releases (eg, Ministry of Health [28,29]). Further, historical data were available for download, and some of them are presented in Figure 1, where they are combined with historical data on the number of active COVID-19 cases in New Zealand on the COVID-19 data portal from Stats NZ, New Zealand’s official data agency [30]. A more detailed graph of the number of active COVID-19 cases in New Zealand, distinguishing import-related and locally acquired cases, can be viewed at the Ministry of Health website [31]. The history of COVID-19 alert levels in the Auckland region—Auckland is the biggest city in New Zealand; the population of the Auckland region centered on Auckland is 1.6 million, about one-third of the total population of New Zealand [32]—is also shown in Figure 1, based on a document released by the New Zealand government [33]; Alert Level 4 corresponds to a lockdown with substantial restrictions on movement, while Alert Level 1 suggests heightened vigilance, but very few restrictions.

As seen in Figure 1, the NZ COVID Tracer app was introduced at the end of the first lockdown, which covered the whole of New Zealand, including Auckland [33], and received very little acceptance over June and July, while the country remained at Alert Level 1. Nonetheless, on August 12, 2020, a COVID-19 case with unknown source was discovered in Auckland, resulting in the alert level being raised to Alert Level 3 in the Auckland region and to Alert Level 2 in the rest of the country. This prompted a steep increase in the use of the NZ COVID Tracer app, with the daily number of QR scans growing by two levels of magnitude. However, the level of use decreased considerably once the country returned to Alert Level 1, although it remained considerably higher than before the second lockdown. Relatively high levels of active cases in October and November were almost exclusively imported cases, reflecting the growth of the pandemic overseas [34], and were not associated with higher use of the app.

New Zealand’s COVID-19 outbreak response, as assessed in October 2020, has been recognized as successful [19]. However, in spite of the growth in adoption over the second lockdown in Auckland, the potential of the NZ COVID Tracer app in contributing to this response was not fully realized. As of November 13, 2020, even though 2.3 million users—almost half of the population of the country—were registered with the app, fewer than 1 in 6 of them were using it daily [25]. In an incident in Auckland involving a COVID-19 case visiting business premises on November 7, 2020, the number of potential contacts who could be traced via the app was very low, prompting the Ministry of Health to issue an appeal to citizens to use the app more [35]. In November 2020, improving user engagement with the NZ COVID Tracer app remained a problem for New Zealand.

### Semistructured Interviews

The semistructured interview guide (Multimedia Appendix 1) was based on the UTAUT framework [11,12] and focused on effort expectancy (ie, effort associated with using the app), social influence (ie, the extent to which important others are perceived as encouraging the use of the app), facilitating conditions (ie, help available), and habit. Following Walrave et al [11], privacy concerns also received focus. Further, the interview guide emphasized perceived severity of COVID-19 (ie, the perceived consequences of being infected) and perceived susceptibility to COVID-19 (ie, the perceived likelihood of getting infected), concepts borrowed from the protection motivation theory (PMT) [36,37]. The benefits of using a mobile contact tracer app were explored at several levels, following Altmann et al [13], distinguishing benefits to the individual, the family, and society in general. Further, the self-reported patterns of use and the associated experiences were explored in detail, focusing both on current use and on how the approach to using the app by the respondent has changed over time. Moreover, the respondents were asked to project how they are anticipating using the app in the future. Respondents were allowed to deviate from the framework suggested by the interview guide, for as long as the interview remained overall relevant to the research questions of the study.

Participants were recruited using an advertising campaign on Facebook targeting Auckland region residents aged 18 to 64 years (55.19% of the New Zealand population are Facebook users [38]). The campaign invited users of the NZ COVID Tracer app to contribute to the fight against COVID-19 by granting an interview. Further, the participants were entered into a draw to win a token prize. All individuals meeting the criteria who expressed interest in being interviewed were interviewed until the desired sample size was reached; thus, a nonprobability consecutive sampling strategy was used. Following Braun and Clarke [39], the sample size was based on the sample sizes found to be sufficient to answer research questions in similar studies, such as Wessels et al [15] and Byambasuren et al [40], and on pragmatic considerations, such as the ability of the researchers to analyze the resulting volume of data within a reasonable time. The interviews were conducted by the first author over Zoom in late October and early November 2020. The interviews were transcribed in full for analysis.

### Analysis

Thematic analysis of interview transcripts was conducted following Braun and Clarke [39]. Both deductive and inductive approaches were used, with deductive coding drawing from the UTAUT and the PMT. Following Braun and Clarke, concepts drawn from the UTAUT and the PMT, as introduced in the previous section, were used as a sensitizing device that was used to attract analysts’ attention to potentially relevant aspects in the data; the aim was to understand user experiences and drivers of app use, rather than to test the UTAUT or the PMT. NVivo 12 (QSR International) was used for coding.

Both coauthors analyzed the data. Both researchers have higher degrees in information technology–related disciplines, with the first coauthor having a stronger technical background and the second coauthor having a background in medicine. Because of the difference in backgrounds, the researchers provided complementary perspectives. The researchers analyzed the data independently, periodically integrating the findings, and resolved differences via discussion.

### Ethics

Following the university’s ethics procedures, a low-risk notification was filed. Participants were informed in writing of their rights, such as the right to withdraw from the study at any point and the right to ask questions about the study. After receiving this information, the participants gave consent in writing.

## Results

### Participants

Interviews were conducted with 34 residents of the Auckland region, with interview durations ranging from 15 to 50 minutes (mean 23, SD 8.9; median 21.4). Participants (Table 1) ranged in age from those in their late teens to those in their early sixties. Even though about half of the participants identified as White New Zealanders of European origin, different ethnicities were represented, including New Zealanders of South Pacific, Indian, Middle Eastern, South American, and Southeast Asian descent. Out of 34 participants, 2 (6%) identified as Māori (Indigenous New Zealanders) and 1 (3%) was a temporary visitor from Europe stranded in New Zealand because of the COVID-19 pandemic (Participant #4). A broad range of careers was represented, from top-middle management to health support work and charity work. Out of 34 participants, 1 (3%) was a female homemaker and 1 (3%) was retired. Likewise, educational backgrounds ranged broadly, from high school completion to master’s degrees. Out of 34 participants, 2 (6%) were unemployed (Participants #1 and #2), having recently lost their jobs because of the pandemic.

**Table 1:** Characteristics of the participants.

Participant No.	Gender	Age range (years)	Ethnicity	Education	Occupation
1	Male	18-29	NZ European^a^	Undergraduate diploma	Sales
2	Female	40-49	NZ European	Bachelor’s degree	Pilot
3	Female	40-49	Macedonian	Master of Business Administration	Data scientist
4	Male	18-29	Caucasian	Master’s degree	Biologist
5	Male	50-59	NZ European	Master’s degree	Training provider
6	Female	50-59	Latin American	Master’s degree	Interpreter
7	Male	30-39	Māori	Bachelor’s degree	Support worker
8	Female	40-49	NZ European	Nursing degree	Nurse
9	Male	40-49	Middle Eastern	Master’s degree	Account manager
10	Female	50-59	NZ European	Postgraduate degree	Teacher
11	Female	40-49	NZ European	Postgraduate degree	Head of compliance
12	Female	50-59	NZ European	Master’s degree	Homemaker
13	Female	60-64	NZ European	Bachelor of Arts	Teacher
14	Female	40-49	NZ European	Postgraduate diploma	Teacher
15	Female	40-49	Scottish	Postgraduate degree	Health manager
16	Female	50-59	NZ European	Bachelor’s degree	Charity worker
17	Female	50-59	Australian	Undergraduate diploma	Customer service
18	Female	50-59	NZ European	Postgraduate	Administration
19	Male	30-39	NZ European	High school diploma	Manager
20	Female	60-64	NZ European	Undergraduate degree	Retired
21	Female	50-59	European	Postgraduate	Health consultant
22	Female	50-59	Indian	Bachelor’s degree	Manager
23	Female	50-59	NZ European	Master’s degree	Teacher
24	Female	60-64	NZ European	Bachelor’s degree	Dental receptionist
25	Male	40-49	Samoan	Trade certificate	Human resources
26	Male	18-29	Korean	High school diploma	University student
27	Female	30-39	European	Tertiary certificate	Manager
28	Male	50-59	Scottish	High school diploma	Business owner
29	Female	40-49	European	University diploma	Sales
30	Male	18-29	NZ European	High school diploma	Customer service
31	Male	40-49	NZ European	Master’s degree	Local government
32	Female	40-49	NZ European	Postgraduate diploma	Health manager
33	Male	30-39	Māori	High school diploma	System engineer
34	Female	50-59	Indian	Postgraduate degree	Travel agent

^a^NZ European: White New Zealanders of European origin.

### Themes

The thematic analysis resulted in five major themes: perceived benefits, patterns of use, privacy, social influence, and need for collective action. These themes are depicted along with the underlying subthemes and codes in Table 2. The content of the themes is presented in detail in the following sections.

**Table 2:** Major themes and the underlying subthemes and codes.

Themes and subthemes		Codes
**Perceived benefits**		
	Society	Contact tracing Location data available to the government
	Family	Self-isolate
	Individual	Does not reduce risk Peace of mind from protecting others Reduction in uncertainty Better experience in contact tracing Personal diary
**Patterns of use**		
	Use by yourself	Scanning codes Manual entries during a visit Manual entries after a visit
	Involving others	Manual entries to record information about others Complaining to location manager if code not available
	Persistence and changes	Persistence in the face of technical difficulties Maintaining use irrespective of the level of threat Use driven by the level of threat
**Privacy**		
	I don’t care	Nothing to hide The information is already out there Others care
	I care, but I comply	Benefits outweigh privacy concerns
	Improvements that would reduce privacy	Recording information automatically Using GPS location data
**Social influence**		
	Encouragement	Encouraged by others Encouraging others
	Help	Help from others Helping others Seeking help from the internet
**Need for collective action**		
	Citizenship	Trust in New Zealand government Civic responsibility
	Frustration	Ignoring conspiracy theorists Frustration by others’ lack of use

### Perceived Benefits

For most of the participants, the most prominent benefit of the app was supporting contact tracing in the context of controlling the pandemic in a broad sense:

Limits the damage and the spread of the virus drastically.

Participant #4

Feels like a very easy habit to maintain and a very small price to pay, because I can absolutely see the value of having a very quick easy method of tracking people.

Participant #11

If the app is doing what it says it’s doing, then you know, one click, and everybody knows, and you’ve captured the problem, and you know that much faster, and we don’t have to go into the stress of this lockdown business again.

Participant #22

Benefits to the family resulting from the individual being able to self-isolate early if at risk of infection were also mentioned:

It definitely gives me peace of mind because I have young children; I obviously never want to put them in harm’s way.

Participant #27

I better remain cautious because of my wider family. My twin sister...if she caught it she would probably die.

Participant #24

Specific immediate health benefits to the individual using the app as well as greater likelihood to be diagnosed early and, thus, to receive early treatment, resulting in better prospects for the individual, were often not clear to the participants:

I wouldn’t say I’m protecting myself. Because probably it doesn’t reduce my risk in any way.

Participant #20

It can’t prevent me from catching COVID, I would say it probably more protects the people around me.

Participant #30

Some of the participants identified reduction in uncertainty as a benefit to the individual using the app (ie, if you are infected, you are likely to know about it faster if you use the app):

I can see the benefit if it happens that I ended up being in contact with somebody that’s got it. I would rather know quicker.

Participant #27

I feel like it’s protecting me by keeping me in the know.

Participant #21

Another individual benefit suggested by the participants was the presumed better experience for the individual that was contract traced in the event contact tracing becomes necessary:

If I get sick, I can concentrate on getting well and I can leave contacting people to the government trackers who are paid to do their job.

Participant #2

I can instantly track back where I’ve gone, and I can provide that information rather than trying to think back, “Oh where was I, did I do that...?” It’s all there.

Participant #31

Further, some of the participants found benefits that are not associated with COVID-19 virus control. For them, the app acted as a diary making it easy to recollect where they have been:

And it’s quite good for me too, I find, because sometimes I forget where I’ve been. And I look at my app.

Participant #10

...helps me remember where I have been.

Participant #26

Overall, while benefits for contact tracing in the context of protecting society in general arose very naturally in the interviews, benefits to the individual using the app were often mentioned only after specific prompting by the interviewer, and different participants had different views on what they are.

Finally, some of the participants perceived the ability of the government to have access to location data and to conduct research using these data as a benefit, although it was not really a benefit because of the privacy features of the app:

Obviously, the government would know where everyone’s going. So that’s like, you know, we’re helping...It is a good thing the government knows where you’ve been.

Participant #26

It’s giving them data and it’s giving them a platform to start developing what could be needed if this pandemic continues, or if there’s a future one.

Participant #24

### Patterns of Use

Simply scanning Ministry of Health QR codes displayed by businesses was the most common use of the app:

Just scan it sometimes if I’m not in a hurry. I don’t scan it if I can’t be bothered. I try to do it every time. But I’m not religious about it, you know...I suppose it’s just a habit now.

Participant #1

I walk into a building, grab my phone out, and swipe the tracer. It doesn’t really change my life, it’s not that difficult.

Participant #2

I plan out when I’m getting out of the car. I have my phone in my hand. Anyway, and I’ll just open the tracer app. And I just, I just walk by, like, you just hardly even have to stop now.

Participant #21

Manual entries were used when a QR code was not available or could not be scanned easily (eg, it was in an inconvenient location or was laminated, so that reflected light inhibited scanning):

But if they don’t, for some reason, have the QR code available, I always put a manual entry in.

Participant #23

I just look for the QR codes and just record visits. Every now and then, it hasn’t worked and I’ve recorded manual visit, but that doesn’t seem to be happening so much.

Participant #14

Another common use of manual entries was to record visits to locations after the fact, when forgetting to scan the QR code:

I try to not forget, wherever I enter any place. But I’ve also caught myself several times, adding it manually after.

Participant #3

I have forgotten and I’ve moved away. And I remember like half an hour later, I put a manual entry in.

Participant #31

When a QR code could not be found, some of the participants complained to the location manager; others just did nothing:

I’ve asked people if you got a poster and gone and found it.

Participant #5

I just go and tell the management...If you don’t display, I’m not comfortable in coming here...That’s what I do if it’s not available, or not prominently displayed.

Participant #34

When I’m entering the store or going to a place and I see a poster, that kind of reminds me to use it. I must admit, when I haven’t seen a poster, I haven’t used it. It’s not automatic...

Participant #10

If there’s nothing I don’t bother asking.

Participant #1

The app allowed users to enter extra information in addition to scanning a QR code. This was occasionally used to record the presence of others who did not scan for themselves, such as children. Sometimes the presence of others was recorded, possibly without their explicit approval:

If I’ve been with other people, like, particularly if my family have been with me, but they haven’t had the phone with them, like my teenage sons. I’ll put down that they were with me.

Participant #18

...she’s trying to download and it doesn’t work on her phone. So when she and I are together, that’s okay, because I know we have a record that we’re out together. And I’m doing it.

Participant #11

Twice I was with somebody who doesn’t have their phone with them...So I just added their name to mine.

Participant #13

Patterns of use were impacted by updates rolled out by the app developers. For many of the participants, the app was entirely unusable at the beginning (eg, did not scan QR codes well enough or did not scan them at all). One of the participants reported installing and uninstalling the app multiple times, until a version that worked on her phone had been released:

The first couple of versions of it was such crap that each time I would end...I would uninstall it and I scream and shout and say, “I am never putting this back on my phone again.” ...Then finally...I try to use it every day, I mean, if I go out. I try to always remember to have my phone.

Participant #17

Few places I tried to use it, it didn’t work. So I put it away for a bit. I think when we went into the next community transmission and they said, or, you know...They seem to have done some work on it and they were raising again that it was good to use. And then I used it a couple of times and it worked. So I thought, “Okay, if it works, then why not?”

Participant #22

Some of the participants reported considerable resilience in continuing to use the app even while experiencing difficulties scanning the codes or being forcibly logged out of the app and having to recover the password:

Sometimes it’s a little bit slow. And not just starting up. It’s a little bit slow and sometimes forgets my password, and I have to log in again.

Participant #1

The only thing that has been a bit annoying would be that sometimes I was logged out...other than that it was a seamless transition. I mean, being logged out means that I need to figure out which was my password and I’m terrible at that. But other than that, I figured it’s quite good.

Participant #3

Participants could be divided according to how their use of the app related to alert levels. While some of them reported continuing to use the app irrespective of the level of the alert—this behavior tended to be associated with the perception of being highly vulnerable to the virus—others reported less consistent use before and after the second lockdown; this was consistent with the pattern suggested in Figure 1:

I am an asthmatic. I would be considered a high-risk group...As a general rule, every time I see a QR code I scan the app.

Participant #31

I’ve had pneumonia before and I know it’s worse than that...The codes at places didn’t work properly...Halfway through the first lockdown. I actually deleted it. And then once they said that they done a few of the bug fixes and I’ve downloaded it again and I can definitely say that I’ve used it quite a lot since then.

Participant #27

I didn’t use it for the first couple of weeks. Because I think when it first came out, we were at Level 1 already...I think I forgot the timing, but it might have been after the second lockdown. I’ve been using it absolutely consistently ever since and continue to do so.

Participant #11

During the lockdowns I always used the app. I mean, because you know it’s a lockdown...But at the moment, with Level 1, I don’t really use the app. And it’s probably because no one else seems to be, whenever I walk into a place.

Participant #25

Very strong belief in the benefits and the necessity of the app and active steps to encourage others to use the app did not rule out reducing use once the alert level went down:

I was a true supporter to start off with. I’ve scanned wherever I could. I suggested to businesses to go and get it [the QR code for the app]. Made sure that our business got it right away, being a real supersupporter. And then we got over the first wave, we got back to work, there were no cases, so that it sort of died down, I’ve seen businesses removing the scan codes...there were no cases for quite a long time, and my use of the app changed, actually using it a lot less.

Participant #29

### Privacy

Most of the participants did not worry about the app reducing their privacy. Privacy, however, was very prominent in the interviews, with the participants often discussing it with no prompt from the interviewer. Reasons mentioned for not worrying about privacy included the following: (1) the participant has nothing to hide; (2) the participants already perceive themselves as having no privacy as they are tracked via social media, by mobile phone service providers, via transaction records, or by other means; and (3) the participant relies on the app’s privacy features:

I don’t care that they know where I’m at. I don’t think they’d care that much.

Participant #1

I don’t have anything to hide. I’m not cheating anybody.

Participant #2

At the end of the day, it’s like these cameras at your workplace. If you got nothing to hide, you don’t have to worry about it.

Participant #19

...knowing what Google and the likes of Google can do...If someone wants to get something on you, everything is available.

Participant #3

All of us like to tap into free Wi-Fi everywhere we go. So really, there’s a lot of data out there about us. But so, no. No, I don’t care.

Participant #22

I’m not worried about the data that it gathers because that data is mine until it is required.

Participant #31

On the other hand, the existence of others who do care about privacy was often acknowledged, with their preferences mostly accepted as legitimate:

It [the data being recorded] doesn’t bother me. I have a son who’s a lawyer who refuses to...use the app. But not me. It does not bother me.

Participant #16

You don’t have to put in your personal details, because there were a few [employees at work] that had privacy concerns with the COVID app, they are worried about people watching them, and we pointed out to them that you do not need to put any personal details into the app. You don’t have to put in your first and your last name, you could call yourself Mister 123 if you really wanted to.

Participant #19

At the same time, some of the participants expressed negative attitudes toward mainstream and social media discourses overemphasizing privacy issues around the app. One of the participants, when asked about others discouraging her from using the app, pointed at one of the major New Zealand newspapers:

Newspapers, like [name of a major New Zealand newspaper], constantly have articles about how it’s taking away our privacy and stuff like that.

Participant #2

Of the two participants who expressed concerns about privacy, one reported weighing privacy concerns against the benefits of faster contact tracing and deciding that benefits overweigh the risks. The other participant reiterated privacy concerns throughout the interview, but the concerns were not focused on the app and, rather, were about the overall environment, including social media and mobile phone service providers. At the same time, when asked how the app could be improved, the participant suggested an improvement that would reduce, rather than increase, privacy:

I am slightly a conspiracy theorist, but I thought weighing it all up, I felt it was more wise for me to embrace it.

Participant #24

This is this app, this is that app, there is a lot, you are controlling my life...It is annoying that you need to be booking everywhere you go, like keeping a diary of everything. It’s a form of controlling, Facebook is a form of controlling, Google knows where you, whatever, is controlling, you have no privacy...Yes, I want to help the government and things like that, but at the same time this is a bit like Animal Farm [a novel by George Orwell].

Participant #6

You go to places, you need to park your car. Maybe integrate with your car parking...

Participant #6

Many participants suggested improvements that would reduce privacy (eg, recording visits automatically using wireless technology, using GPS to track app user location, or using wireless technology to automatically detect and record the proximity of others). None of the participants suggested changes to the app that would increase privacy.

### Social Influence and the Need for Collective Action

Overwhelmingly, the participants expressed high levels of trust in the New Zealand government. Often, the fact that information comes from the government was a criterion of its trustworthiness. Using the app was seen as their civic duty and a way to be a good citizen of New Zealand. Moreover, some of the participants framed patterns of behavior in terms of “good” and “bad.” A phrase introduced by the Prime Minister and repeatedly used in communications about the pandemic by her and other officials was commonly mentioned: “the team of the five million” [41,42]:

I go straight to the New Zealand government COVID-19 website. I try to stay away from the internet.

Participant #28

I feel like I should do it [use the app]...just to be a good citizen of New Zealand united team of 5 million and all of that.

Participant #1

I feel like you kind of need to set the example...ultimately, if I get sick I would feel that it is my civic responsibility to make sure that anyone who was near me, came in contact with me, would get the health care that they might need.

Participant #33

In the beginning there was a lot of talk about it in the news, and people saying, “Oh no, that’s stealing all your personal data.” But I don’t see it this way at all. I think this is good data to be used in a good way.

Participant #25

However, trust in the government was not a prerequisite for using the app. One of the participants, a manager in an industry that was highly critical in maintaining the functioning of the city during lockdowns, expressed very low levels of trust in the government, even suggesting that the government purposefully distorted some of the information related to the pandemic; at the same time, he reported not only using the app but also ensuring that it was installed on mobile devices used by employees, as well as establishing procedures to ensure that visitors to company premises used the app:

I think there’s a lot covered to avoid panic. So, yeah, yeah, it’s hard to trust.

Participant #19

The flow of social influence was rather complex, involving multiple actors. Participants reported being encouraged by others to use the app. Further, for some, using the app was a requirement at their workplace. Participants also reported encouraging others, in face-to-face settings and online:

My parents actually...said, “You should probably get it [the app].” And I said, “Yeah, yeah.”

Participant #26

I installed it I when I still had a job...It was a requirement for me as part of my job to use it. But then when I lost my job, I became more flexible with it. Like, it’s not a requirement. Now for me, it’s just something that I grew in, it became a habit.

Participant #1

My husband, he does use it a lot. Whenever we go out, and if sometimes I rush going somewhere, he just stops me and says, “Just scan it.” He always reminds me and encourages me to be more vigilant...My friends, I just told them to install it, maybe they have, I do not know...When the app was first introduced, I sent messages to a lot of my contacts.

Participant #34

Businesses displaying the codes occasionally encouraged app use; in their turn, some of the participants actively engaged with businesses when a code could not be found or was unusable, as already highlighted in the Patterns of Use section:

Now [after the second lockdown ended] I’m not really using it, no. I only use it if the shopkeeper asks me to use it and then I will say, “Of course I’ll use it.”

Participant #26

We do actively, when we have customers coming to pick items, my receptionist will say...“Can you scan in please?” We literally say it to everyone who walks in...To be honest, people don’t scan when they are coming in, the instance you say, “Would you mind scanning,” nobody’s ever thrown anything back at us, they just say, “No worries,” they do it.

Participant #19

Some of the participants relied on internet-based resources, such as the Ministry of Health website, when having problems using the app. Help was not always readily available:

...just go to the [Ministry of Health] website for help.

Participant #25

I look at the Ministry website...

Participant #8

...and I’m going to COVID-19 website and I cannot even find where the bloody test centers are.

Participant #9

I even tried to contact someone and say, “You know, it’s not working.” And then I figured out, I guess, you know, it’s not working. So they’re getting too much communication...but once it has started working, like once I started using it the second time round, haven’t had any need to contact anyone for help.

Participant #22

Family members and colleagues may have been a readier source of help:

I’ll ask my 21-year-old son. He is quite tech savvy. So I utilize expertise inside my family.

Participant #28

I would ask my husband.

Participant #27

I was coming back by bus...and it was not scanning...somebody was sitting next to me, my colleague...I passed my phone to her, and she scanned it for me, because she was a little bit closer.

Participant #34

Further, strangers helped each other:

I was in the supermarket and then was having difficulty. And the guy said, “Oh, you know, you need to turn on the camera.”

Participant #6

I do see a lot of young people helping older people use it.

Participant #8

I’ve helped a couple of people to download it.

Participant #21

The existence of “conspiracy theorists” raising, from the perspective of the participants, unreasonable or untrue privacy concerns, was occasionally acknowledged. Although one of the participants described himself as a “conspiracy theorist,” he still used the app, judging that the benefits were greater than the risks, as introduced in the Privacy section:

...some think that the COVID-19 scanning app was taking the wrong information on each individual to hand more information to the government power. I don’t believe that myself. Those conspiracy theorists inside my own family unit, I took it with a grain of salt. So meaning...I believe in the app.

Participant #28

My mother-in-law, she’s saying, you know, “Don’t use that, don’t install it, because they’re tracking all your data and everything.”...I’m ignoring her for a lot of things.

Participant #18

Many of the participants were concerned about the behavior of others in using the app and in reducing the risk of COVID-19 spread in other ways. The realization that protecting the country from the pandemic depended on collective action was rather strong. Often, rather than expecting the authorities to improve technological capabilities or ease of use of the app, the participants highlighted the need to encourage its broader use:

I find it quite frustrating going to places and seeing people around me who, you know, are walking on without even bothering to scan.

Participant #23

I think New Zealand’s getting very complacent.

Participant #2

My friends or family don’t use it. Full stop.

Participant #19

My concern is that we have a lot of people just disregarding the impact of COVID.

Participant #8

I think, some shops are deliberately making it [the QR code] hard to find.

Participant #5

## Discussion

### Principal Findings

The main contribution of this study of adoption and use of a mobile contact tracing app is that it is based on data reflecting real user experiences, rather than on perceptions of individuals who are yet to use such an app. Prior studies predicting mobile contact tracing app adoption and use relied on data obtained from nonusers.

The results of this study are consistent with the finding by Trang et al [8] that perceptions of benefits for society as a whole are likely to drive the use of a mobile contact tracing app. However, the results also suggest that such benefits are mainly relevant when the level of threat to society is high. For many individuals, but not all, the logic of taking individual action to protect the society on which the individuals depend is powerful enough to drive sustained use only when the threat to the society is salient enough.

The results of our study are consistent with Altmann et al [13] in suggesting that trust in the government helps to promote mobile tracing app use. Nonetheless, the finding by Altmann et al [13] that concerns about government surveillance are very important were not confirmed by our study. This may be, in part, because our study covered only the users of the app, who were likely to fall into the “advocates” category following Trang et al [8]. Assuming the participants of this study were “advocates,” the finding that privacy did not matter for them is consistent with the results by Trang et al [8]. The results of our study are consistent with Wiertz et al [9], who also found little evidence that privacy is highly relevant, as well as with the body of literature on the privacy paradox [43], which suggests that in actual use, users are prepared to trade their privacy even for rather small benefits.

The NZ COVID Tracer app was designed in such a way that its use, or nonuse, was highly visible. Thus, social influence, found to have an effect by Walrave et al [11], could be highly influential. Nonetheless, for most of the participants, social influence by peers appeared to play a secondary role in driving their app use. Indeed, some of them continued to use the app while surrounded by nonusers. For them, social influence was coming from the government, not from the peers. At the same time, the results indicate that organizations may be effective in promoting the use of the mobile tracing app by their employees: employees who are not users are likely to comply to become users, rather than resist.

Our study found no indications that an app overseen by an independent entity, rather than by the government, would be better accepted or used more, and in this respect, our results did not confirm the results by Wiertz et al [9]. Indeed, the discourse by the app users around good citizenship and civic duty as reasons for using the app suggested that oversight by the government was a good choice in the New Zealand context. Nonetheless, this conclusion has to be confirmed by a study of nonusers of the app.

The study by Walrave et al [11] did not find effort expectancy to be an important factor. Our results were consistent with this finding. Determined users of the app were prepared to persist in the face of technical difficulties. This is not to suggest that effort expectancy is irrelevant; however, there was little evidence to suggest that, after the initial bugs were fixed, making the app even more effortless to use would result in significantly higher adoption and use.

The implications for practice are that appeals to civic responsibility are likely to drive the use of a mobile tracing app under the conditions of high threat, as citizens “rally around the flag.” Under the likely scenario of COVID-19 remaining endemic and requiring ongoing vigilance over the long term, other mechanisms promoting the use of mobile tracing apps may be needed, such as “nudging” [44] (eg, offering incentives). Further, the results suggest that privacy is not an important concern for many users. Having access to more detailed information faster would benefit contact tracing, enabling faster isolation of probable cases and, thus, better control of the pandemic. Therefore, compared with a mobile tracing app with uniformly restrictive privacy features, an app with flexible privacy settings allowing users to set their optimal levels of privacy—thus allowing users who are less concerned about privacy to opt in to provide more detailed information faster—may be more appropriate.

The value of comparing responses between countries in informing decision making in the COVID-19 pandemic has been highlighted by Pearce et al [45], who characterized health policy responses in different jurisdictions as “numerous natural experiments in progress” (page 1059 in Pearce et al [45]). The case of New Zealand is particularly valuable in this respect because it presents an example of a successful response [46,47] achieved in a country with western political culture [48]. As such, the New Zealand experience in managing the pandemic has received a lot of attention in international literature [49-56]. Our study contributes to this body of literature by focusing on the experiences of the users of the NZ COVID Tracer app. However, the results of this study, as well as of other studies of the New Zealand experience in managing the COVID-19 pandemic, cannot be mechanically applied to other contexts. Rather, as for most qualitative studies, the process of case-to-case transfer [57] should apply: the readers and the consumers of the research should compare their context of interest to the New Zealand context and judge the extent to which the findings apply to their situation (page 1453 in Polit and Beck [57]). A broad description of the New Zealand context as it applies to the management of the COVID-19 pandemic is given by Jefferies et al [54], who assert that the New Zealand response to COVID-19 “has international relevance, particularly for other island nations, high-income and western settings” (page e613 in Jefferies et al [54]). Further, aspects of the context immediately relevant to the research questions of our study, such as the app design and the way it was introduced, relying on persuasion rather than on mandates, are described in the initial sections of this paper.

### Conclusions

Appeals to civic responsibility are likely to drive the use of a mobile contact tracing app under the conditions of high threat. Under the likely scenario of COVID-19 remaining endemic and requiring ongoing vigilance over the long term, other mechanisms promoting the use of mobile contact tracing apps may be needed, such as offering incentives. As privacy is not an important concern for many users, flexible privacy settings in mobile contact tracing apps allowing users to set their optimal levels of privacy may be appropriate.

## Appendix

Multimedia Appendix 1 Interview guide.

## Abbreviations

PMT: protection motivation theory
QR: Quick Response
UTAUT: uniﬁed theory of acceptance and use of technology
