# Supplementary material for: User Experiences of the NZ COVID Tracer App in New Zealand: Thematic Analysis of Interviews
Source: JMIR Mhealth Uhealth. 2021 Sep 8;9(9):e26318. doi: 10.2196/26318 (PMC8428377; doi:10.2196/26318)
Supplement: Multimedia Appendix 1 [file mhealth_v9i9e26318_app1.docx]

## Multimedia Appendix 1

# Interview guide

1. In which ways did your life change since the onset of the COVID-19 crisis?
2. In which ways can the CORONA-19 pandemic affect you in the future?
3. CORONA-19 disease - is it dangerous?
4. What are your sources for information about CORONA pandemic? (Prompt: How do you distinguish, which sources to trust?)
5. How did you learn about the tracer app?
6. Would you describe, how you normally use the COVID-19 tracer app? (Prompts: Do you use it every time a poster with QR code is available? What do you do if a poster is not provided? Do you enter any other information, in addition to scanning the QR code?)
7. How did your approach to using the app change over the period since you have installed it?
8. How do you expect to be using the app in the future?
9. From your perspective, what are the benefits of using the app? (Prompts: Benefits to you personally? Your family? Your community? The country?)
10. Do you have privacy concerns about using the app?
11. Which problems did you experience using the app?
12. What do you do when you need help using the app?
13. Did you ever help others using the app?
14. In which ways did other people encourage you or discourage you in using the app?
15. Did you do anything to encourage others to use the app?
16. How can the app be improved?
17. Are there alternatives to using the app?
